# WASP: a Web-based Allele-Specific PCR assay designing tool for detecting SNPs and mutations

**DOI:** 10.1186/1471-2164-8-275

**Published:** 2007-08-14

**Authors:** Pongsakorn Wangkumhang, Kridsadakorn Chaichoompu, Chumpol Ngamphiw, Uttapong Ruangrit, Juntima Chanprasert, Anunchai Assawamakin, Sissades Tongsima

**Affiliations:** 1 Biostatistics and Informatics Laboratory, Genomics Institute, National Center for Genetic Engineering and Biotechnology, Thailand Science Park, Pathumtani, Thailand; 2 Division of Molecular Genetics, Department of Research and Development, Faculty of Medicine Siriraj Hospital, Bangkok, Thailand

## Abstract

**Background:**

Allele-specific (AS) Polymerase Chain Reaction is a convenient and inexpensive method for genotyping Single Nucleotide Polymorphisms (SNPs) and mutations. It is applied in many recent studies including population genetics, molecular genetics and pharmacogenomics. Using known AS primer design tools to create primers leads to cumbersome process to inexperience users since information about SNP/mutation must be acquired from public databases prior to the design. Furthermore, most of these tools do not offer the mismatch enhancement to designed primers. The available web applications do not provide user-friendly graphical input interface and intuitive visualization of their primer results.

**Results:**

This work presents a web-based AS primer design application called WASP. This tool can efficiently design AS primers for human SNPs as well as mutations. To assist scientists with collecting necessary information about target polymorphisms, this tool provides a local SNP database containing over 10 million SNPs of various populations from public domain databases, namely NCBI dbSNP, HapMap and JSNP respectively. This database is tightly integrated with the tool so that users can perform the design for existing SNPs without going off the site. To guarantee specificity of AS primers, the proposed system incorporates a primer specificity enhancement technique widely used in experiment protocol. In particular, WASP makes use of different destabilizing effects by introducing one deliberate 'mismatch' at the penultimate (second to last of the 3'-end) base of AS primers to improve the resulting AS primers. Furthermore, WASP offers graphical user interface through *scalable vector graphic *(SVG) draw that allow users to select SNPs and graphically visualize designed primers and their conditions.

**Conclusion:**

WASP offers a tool for designing AS primers for both SNPs and mutations. By integrating the database for known SNPs (using gene ID or *rs *number), this tool facilitates the awkward process of getting flanking sequences and other related information from public SNP databases. It takes into account the underlying destabilizing effect to ensure the effectiveness of designed primers. With user-friendly SVG interface, WASP intuitively presents resulting designed primers, which assist users to export or to make further adjustment to the design. This software can be freely accessed at .

## Background

One of the results from human genome project is the use of human variation data called single nucleotide polymorphism (SNP). Such polymorphisms including rare ones such as mutations reflect how we have different genetic responses to the environment as well as predispositions to certain diseases and varying effects to drug treatments. These variations, therefore, play a crucial part in many studies including population genetics, molecular genetics and pharmacogenomics. Consequently, to capture the underlying variations, many genotyping technologies have been proposed, such as *restriction enzyme *assay, *hybridization *assay, *ligation *assay, *invasive cleavage *assay and *allele-specific polymerase chain reaction *assay. Each of these techniques has both advantages and disadvantages. In this work, we focus on a simple and cost-effective protocol, namely the allele-specific polymerase chain reaction or AS-PCR genotyping technique. This assay protocol were adopted by many recent studies, for instance, the detection of common alleles of cytochrome P450 that mediates different level of drug metabolisms [1], the screening of mutations responsible for beta-thalassemia [2], and the evaluation of using a set of SNPs as genetic markers of a disease [3].

In literature, AS-PCR is also known as amplification refractory mutation system (ARMS) [4]. This technique is a quick and dependable genotyping protocol that requires minimal instruments found in most laboratories. It is based on the extension of primer only when its 3'-end is a perfect complement to the allele present in the input sample. Thus, if a single base polymorphism occurs, the genotyping results can be observed by simply comparing the length of PCR products. Several automated bioinformatics tools are available for specifically designing AS primer, such as, Tetra-primer [5], Primo SNP [6], and a commercial software *Visual-*OMP [7]. However, none of the available tools offer an integrated input system that users can retrieve markers to be genotyped from within the program framework. Furthermore, the resulting AS primers from these programs may not be specific enough; that is only one mismatch at the terminal base may not discriminate the amplification results from *wild *and *mutant *types since the extension of the allele-nonspecific primer may continues if terminal mismatching has weak-destabilizing effect [8].

To address these difficulties a SNP database and intuitive graphical interface should be integrated with the AS primer design tool while primer-destabilizing condition must be considered to ensure effectiveness of primer results. Consequently, a novel tool, WASP (Web-based Allele-Specific PCR Primer designing tool), is presented in this paper. This tool automatically generates well-calibrated discrimination conditions for AS-PCR assay. By adopting the protocol, our proposed tool can increase the AS primer specificity during PCR reaction by creating a mismatch on the *penultimate *(second to the terminal) base of the AS primer. WASP utilizes a local SNP database, comprising SNPs from dbSNP [9], HapMap [10], and JSNP [11], to search and retrieve input SNP information from within the tool. Therefore, this eliminates extra steps of gathering SNPs from different websites and copying/pasting to a primer-designing tool. Through a unique SVG graphical user interface, WASP offers a primer design for multiple SNPs that store in public databases. Note also that WASP calculates AS-PCR assay for genotyping SNPs, which are not in this database by providing flanking sequences as well as the target SNP(s) to the program. Optionally to optimize PCR experiment, standard PCR conditions can be modified through the primer-parameters. If design conditions are met, AS primers and their *common *oligo-primers will be graphically visualized or otherwise displayed the reasons why they cannot be designed. Once resulting AS primer set is obtained, these primers can also be verified for their global uniqueness by running through an *in silico *PCR [12].

## Implementation

**Figure 1:**
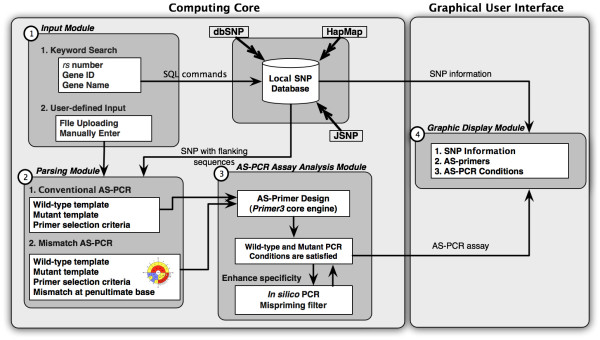
**WASP computational flow diagram**. (1) the input module, (2) the parsing module, (3) the AS-PCR assay analysis module and (4) the graphic display module.

WASP is a web application constructed using Ruby on Rails Framework. The application is integrated into the local SNP database (shown in Figure 1) running MySQL database server. This database frequently collects public SNP information and related reference data from public SNP databases. At the time of writing current version of these databases are dbSNP build 126, HapMap public release 20 and JSNP release 28. The computing core runs within UNIX environment comprising four modules: 1) *the input module*, 2) *the parsing module*, 3) *the AS-PCR assay analysis module *and 4) *the graphic display module*.

### 1) Input module

The purpose of this module is to read in target SNP(s) or mutation(s) that users want to construct an AS-PCR assay. WASP uses two alternative input formats, namely keyword and user-defined input. First, the keyword search is designed for looking up related SNP information such as possible alleles and their corresponding flanking sequences. The allowed keywords can be Gene name and reference SNP *rs *number. The input module creates a set of SQL queries to retrieve underlying information such as the 5' and 3' flanking sequences and the target alleles of SNP; then pass the resulting information to the parsing module to further prepare inputs for Primer3 program (see *AS-PCR assay analysis module*). For visualization and selection of SNPs to be genotyped, an SVG-based graphical view of SNP locations will also be displayed once a gene is selected.

**Figure 2:**
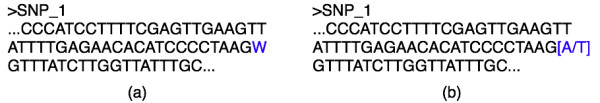
**WASP sample input**. (a) IUPAC ambiguity code and (b) Bracketing code format

The second input format is constructed to facilitate the AS primer design for *novel *SNPs and/or mutations. The target variations and their corresponding 5' and 3' flanking sequences must be entered to the given text box or upload a file containing the information to the server. Instead of constructing SQL queries described previously, the input data will be converted to the internal forms by the parsing module and later sent to the AS primer analysis module. For example, Figure 2 demonstrates two possible sample inputs that WASP would except to calculate the corresponding AS primer(s). The input format for this section comprises 5' and 3' flanking sequences or a target variation, which is described in IUPAC (Figure 2a) or bracketing format (Figure 2b). Note that for both inputs (querying from database and manually entering), their primer conditions can be modified such as primer length, melting temperature (Tm), GC content for primer oligos and other standard primer design parameters.

### 2) Parsing module

**Figure 3:**
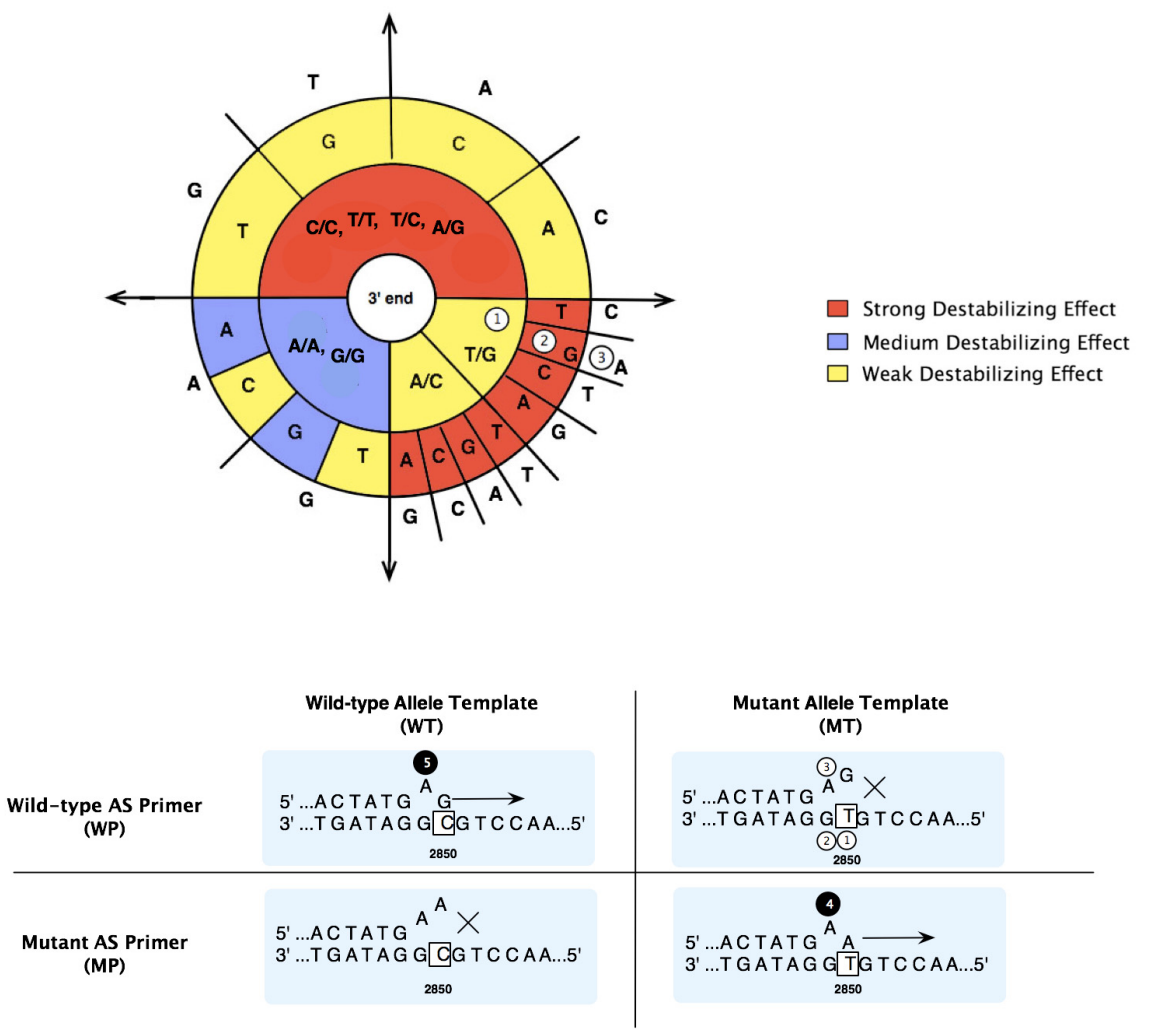
Graphical view of destabilizing effect adapted from ARMS done by Little [7].

This module is responsible for transforming the underlying variation information and their primer conditions to PCR template sequences and primer selection criteria that are used in the Primer3 program [13]. Both *wild *and *mutant *type templates are generated separately, however, they must share the same primer selection criteria in order to be performed in single PCR reaction. The main contribution of this module is the preparation of an *additional deliberate mismatch *at the penultimate base of AS primers to increase the reaction specificity [8]. For instance, if there exists a weak destabilizing at the terminal mismatch, it is likely that such a mismatch is not enough to stop the extension by *Taq* polymerase. To increase the discriminative power, hence, an additional mismatch with a strong destabilizing effect should be introduced at the penultimate base of the AS primers. Figure 3 demonstrates the decision criteria, based on the destabilizing conditions, in which the underlying additional mismatch should be added to AS primer. The areas shaded in red and yellow respectively represent the strong and weak destabilizing effect of any two nucleotides while blue ones imply the medium effect. Starting from the center of this figure (3' end of AS primer) and working outward, we can decide which base (the outermost base of this diagram) to be placed on the penultimate position of the designed AS primer. An example situation showed under the diagram, AS primer was designed for genotyping C>T SNP at +2850 locus of the CYP2D6 gene [14]. The polymorphisms, C (wild-type) and T (mutant), are displayed in the box on the template sequence (3' to 5' direction). On top of each template, primer extension direction is indicated by → whereas × marks a non-extended scenario. Note that nucleotides in the AS primers that are not complementary to the target appear afloat from the primer oligos. Basically, four binding scenarios could happen in a single AS-PCR experiment: WP-WT, WP-MT, MP-WT and MP-MT. To design the AS primers the above polymorphism, wild type and mutant AS primers must have respectively bases G and A at the 3' terminus; thus gel electrophoresis bands can be observed when successful hybridization occurs.

In order to enhance reaction specificity, a deliberate mismatch is introduced at the penultimate base. Using the above diagram, we first consider wild type primer with the case WP-MT in which T/G () presents a weak-destabilizing mismatch (yellow). We then look at the penultimate location to observe the base on the template sequence, which is G () in this case. According to the diagram only nucleotide A () should be put next to the terminal base in both AS primers to ensure the stronger destabilizing (red). Note here that the same additional mismatch is also introduced to mutant primer. If the terminal base is not mismatching, the additional mismatch on the penultimate base ( and ) of the primer will not prevent the extension.

### 3) AS-PCR assay analysis module

Three oligo primers are to be produced by the parsing module, namely wild-type, mutant and common primers. The AS-PCR assay will be conducted in two parallel experiments: one is "wild+common" primer experiment (WC) and the other is "mutant+common" primer experiment (MC). The results will be interpreted from each experimental result. Three scenarios are to be detected, which are homozygous wild type, heterozygous and homozygous variant type respectively. A band from WC but none from MC tube entails homozygous wild type. On the other hand, single band on MC but none from WC tube implies homozygous variant type. If this kind of band appears on both WC and MC tubes, being heterozygous will be inferred.

With the above AS-PCR assay, the core engine of this module is the Primer3 program with some modification to make Primer3 fit the needs of AS primer designing. Two criteria must be considered for this AS-PCR assay. First, AS primers must be used with their *common primer *in the same reaction. Secondly, the reaction conditions should promote the discrimination of wild and mutant reactions. In this module, optimal *primer candidates *will be computed. Both wild and mutant primers are examined if they follow given AS-PCR assay criteria. Once satisfied, each primer pair will be aligned against the human genome reference sequence stored in the local database using *in silico *PCR. This technique utilizes BLAT (BLAST-Like Alignment Tool) to rapidly search for all possible PCR products in the target genome that complement with both directions of PCR primers. In our case, the input of *in silico *PCR is a pair of optimal primer candidates and the output returns PCR products if any. This feature ensures that the global uniqueness of a primer pair can be achieved.

### 4) Graphic display module

**Figure 4:**
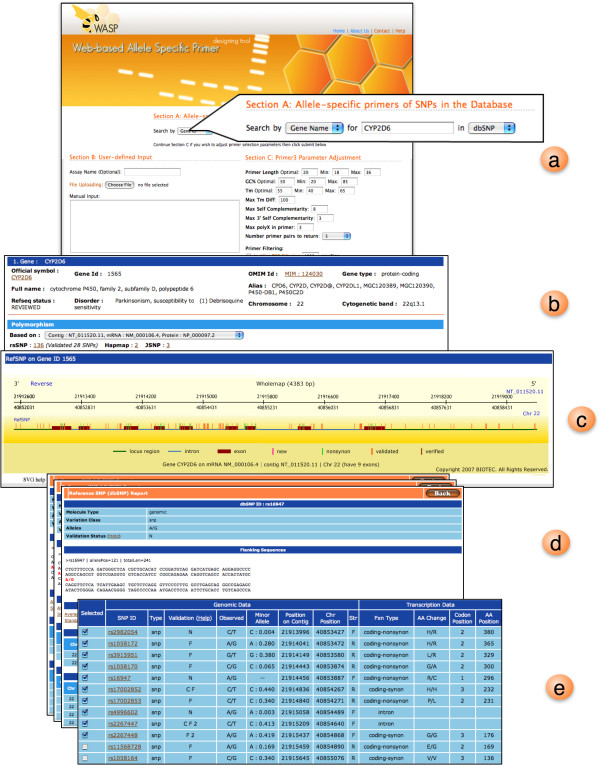
**WASP SVG graphical user input interface**. (a) WASP first page containing three input sections: 1) keyword input for local SNP db, 2) user-defined input and 3) AS primer parameter adjustment. (b) CYP2D6 gene output resulting from using CYP2D6 as a keyword search. The general information including gene locus, gene type, OMIM Id and related disease are reported here. Polymorphism related information on CYP2D6 gene is also shown e.g. number of SNPs in CYP2D6 gene according to dbSNP, HapMap and JSNP. (c) Gene structure view of CYP2D6 displayed as SVG image. The top ruler displays positions and a direction of the gene relative to the reference Contig and chromosome respectively. (d) Detailed information of each SNP including flanking DNA sequences, observed alleles, allele frequency, position on Contig/chromosome, amino acid change and its position relative to the transcription. (e) Selectable SNP list for AS primer design. Using the previously described information, users can select a set of SNP from this table for designing AS primers.

The graphic display module is responsible for graphically displaying the query SNPs as well as the designed primer(s). We adopted the scalable vector graphic (SVG) display [15] that allows users to interact with high quality vector image that can be scaled and move around without viewing degradation due to losses in image compression. The construction of such images is performed "on-demand", i.e., no images are pre-calculated and stored. First, for SNP location map (see Figure 4c), relative locations on each gene are computed upon the query to the local SNP database. On SVG enabled web browsers such as Firefox and Opera SVG images can be displayed without extra installation while all other browsers SVG plug-in module must be installed.

**Figure 5:**
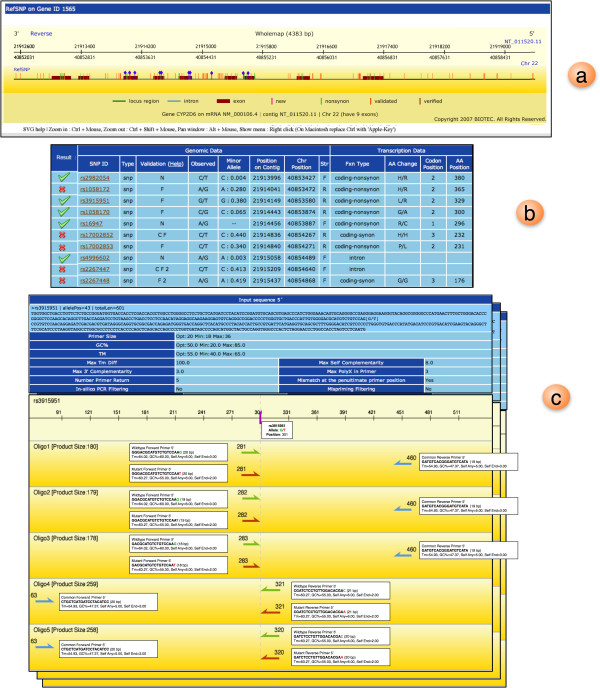
**Graphical output of designed AS primers**. (a) SVG graphical view of selected SNPs shown by blue dots on the SNPs. (b) Table indicates if the selected SNPs have their AS primers successfully designed. (c) By clicking on the checked row in the table, the SVG view of designed AS primers will be displayed. Top 5 oligo primers are displayed for each design where green and red arrows (⇉ or ⇇) show wild and mutant AS primers respectively. The blue arrow (← or →) indicates the common primer. Sizes of the primers are displayed above the arrows. Upon mouse-over action on each primer, the detailed information will be presented in the box next to it.

Second, for displaying AS primers results, the display module waits for results from AS-PCR assay analysis module to be completed. Then the primer information such as orientation, location, and other calculated conditions are converted to SVG image. Here, target SNPs/mutations and the resulting AS primers will be visualized in graphical objects. When moving a mouse over each object, there will be a popup window whose contents show related primer conditions. If no AS primer is reported, only input sequence and original primer parameter conditions will be presented. In this case, some primer selection criteria should be optimized such as varying primer length, Tm, GC content as well as increasing the 5'and 3' flanking sequence length. Figure 4 demonstrates SVG graphical user interfaces described previously using AS primer design for SNPs in CYP2D6. This example demonstrates the case when multiple SNPs on the gene can be selected for AS primer design (Figure 4e). Figure 5 presents primer report (Figure 5a–5b) as well as the SVG output representation (Figure 5c). The results are collectively reported in one continuous page if there are multiple SNPs to be designed. Such results can directly be saved as a text file for future experiments.

## Results and Discussion

Integrating SNP database to WASP makes it possible to display SNP related information to users (as seen in Figure 4b–4d); hence we can use such information to decide which SNP in a given gene to be designed for AS primers. The proposed tool can design more effective AS primers than other available software (Tetra-primer, Primo SNP and *Visual-*OMP) since WASP checks the uniqueness of the primer using *in silico *PCR during the design. Moreover, by incorporating ARMS enhancement protocol to the software, such AS primer results can be even more effective as this technique has been adopted by several recent studies. Note that other existing tools only produce mere primers but not the counterpart "common primer", which needs to have very similar condition to the AS primers. WASP also offers SVG graphical user interface for both SNP selection and AS primer viewing. For SNP selection, WASP allows users to visualize SNPs from a gene structure view (showing intron, exon, 5' and 3' UTR regions of CYP2D6 gene in Figures 4c and 5a). Users need not to construct the input information by accessing other public SNP databases. Finally, unlike other tools, WASP graphically displays location of top 5 output primers as well as their common primers. This kind of outputs should help users tailor their final primers to fit the AS-PCR assay.

## Conclusion

We presented WASP, a web-based allele specific primer design application. The tool conveniently assists scientists in getting SNP information from local SNP database, mirrored from all major public databases, in order to design AS primers for existing SNPs. While current AS-PCR tools such as Tetra-primer, Primo SNP and Visual-OMP, require manual entering SNP information, WASP simplifies the data preparation step by graphically displaying available SNPs to be genotyped and transparently send such information to be computed by Primer3 program. This tool also offers a conventional input for either confirming the information in the database or designing AS primers for novel SNP/mutation.

To improve the specificity of PCR reactions, WASP deploys an *in silico *PCR by searching if a given primer is unique in the genome. Furthermore, WASP offers a missing feature in other primer design tool to improve the effectiveness of the resulting AS primers; that is the introduction of a proper mismatch to the penultimate nucleotide next to the allele specific site in the primer. SVG visualization is integrated into WASP so that primer results can be displayed interactively and intuitively; thus helping users to adjust primer parameters for the negative AS primer outputs. For positive AS primer results, users can export the primer conditions as a text file.

## Availability and Requirements

WASP sever is available for public access at 

WASP requires SVG enabled web browser Opera and Firefox, which are freely available on almost all major Operating Systems , . For Mac OS X, browser such as Safari, Omniweb must have an SVG plug-in installed. The plug-in can be downloaded from . On Microsoft Windows system, a built-in web browser, IE, would also require SVG plug-in to be installed. Without the plug-in rendering all other features will still work except SVG visualization.

## Abbreviations

AS-PCR, Allele Specific Polymerase Chain Reaction

ARMS, Amplification Refractory Mutation System

AS Primer, Allele Specific Primer

SNP, Single Nucleotide Polymorphism

## Authors' contributions

PW and JC wrote the core engine of WASP while KC and UR contributed to the SVG visualization of the tool. CN and ST took care of all local SNP database construction. ST and AA initiated the idea of WASP. ST and PW wrote the manuscript whereas AA reviewed and tested the software.
